# Comparison of raw accelerometry data from ActiGraph, Apple Watch, Garmin, and Fitbit using a mechanical shaker table

**DOI:** 10.1371/journal.pone.0286898

**Published:** 2024-03-29

**Authors:** James W. White, Olivia L. Finnegan, Nick Tindall, Srihari Nelakuditi, David E. Brown, Russell R. Pate, Gregory J. Welk, Massimiliano de Zambotti, Rahul Ghosal, Yuan Wang, Sarah Burkart, Elizabeth L. Adams, Mvs Chandrashekhar, Bridget Armstrong, Michael W. Beets, R. Glenn Weaver

**Affiliations:** 1 Department of Exercise Science, University of South Carolina, Columbia, SC, United States of America; 2 Department of Computer Science and Engineering, University of South Carolina, Columbia, SC, United States of America; 3 Division of Pediatric Pulmonology, Pediatric Sleep Medicine, Prisma Health Richland Hospital, Columbia, SC, United States of America; 4 Department of Kinesiology, Iowa State University, Ames, IA, United States of America; 5 SRI International, Menlo Park, CA, United States of America; 6 Department of Epidemiology and Biostatistics, University of South Carolina, Columbia, SC, United States of America; National Institute of Biomedical Innovation Health and Nutrition, JAPAN

## Abstract

The purpose of this study was to evaluate the reliability and validity of the raw accelerometry output from research-grade and consumer wearable devices compared to accelerations produced by a mechanical shaker table. Raw accelerometry data from a total of 40 devices (i.e., n = 10 ActiGraph wGT3X-BT, n = 10 Apple Watch Series 7, n = 10 Garmin Vivoactive 4S, and n = 10 Fitbit Sense) were compared to reference accelerations produced by an orbital shaker table at speeds ranging from 0.6 Hz (4.4 milligravity-mg) to 3.2 Hz (124.7mg). Two-way random effects absolute intraclass correlation coefficients (ICC) tested inter-device reliability. Pearson product moment, Lin’s concordance correlation coefficient (CCC), absolute error, mean bias, and equivalence testing were calculated to assess the validity between the raw estimates from the devices and the reference metric. Estimates from Apple, ActiGraph, Garmin, and Fitbit were reliable, with ICCs = 0.99, 0.97, 0.88, and 0.88, respectively. Estimates from ActiGraph, Apple, and Fitbit devices exhibited excellent concordance with the reference CCCs = 0.88, 0.83, and 0.85, respectively, while estimates from Garmin exhibited moderate concordance CCC = 0.59 based on the mean aggregation method. ActiGraph, Apple, and Fitbit produced similar absolute errors = 16.9mg, 21.6mg, and 22.0mg, respectively, while Garmin produced higher absolute error = 32.5mg compared to the reference. ActiGraph produced the lowest mean bias 0.0mg (95%CI = -40.0, 41.0). Equivalence testing revealed raw accelerometry data from all devices were not statistically significantly within the equivalence bounds of the shaker speed. Findings from this study provide evidence that raw accelerometry data from Apple, Garmin, and Fitbit devices can be used to reliably estimate movement; however, no estimates were statistically significantly equivalent to the reference. Future studies could explore device-agnostic and harmonization methods for estimating physical activity using the raw accelerometry signals from the consumer wearables studied herein.

## Introduction

Over the past 20 years, device-based assessment of physical activity has improved due to the introduction of wearable monitors, such as accelerometers. Wearable monitors provide device-based estimates of movement and overcome recall and desirability bias that may hamper self-reported measures of physical activity [1, 2]. Best practice recommendations for using accelerometers have shifted over the last decade from traditional activity counts (accelerations per a given epoch) [3] to using raw accelerometry data from accelerometers (i.e., x-, y-, and z-axis accelerometry data in ɡ’s typically collected multiple times per second) to estimate physical activity [4].

Consumer wearables (e.g., Apple Watch, Fitbit, Garmin) are increasingly popular measurement tools for assessing physical activity. Not only are these devices equipped with accelerometers to capture movement, but they are also unobtrusive and designed to be worn on the wrist, targeted for comfort and style, affordable for consumers, rechargeable, waterproof, and can be designed for children [5–8]. Technological advances allow consumer wearables to also frequently have extended battery life (i.e., up to 54 days) [9] and remote data capture and monitoring. For these reasons, there has been a multitude of measurement studies that have explored the validity of physical activity estimates produced by consumer wearables [10, 11].

However, these studies are limited because they rely on estimates of physical activity that are derived from proprietary algorithms developed by the companies that produce these devices (e.g., Apple, Garmin, Fitbit, etc.). This is a key limitation because these algorithms are unavailable for review by researchers [12]. The drawbacks of estimating physical activity based on proprietary algorithms are that it is unclear whether best practice recommendations were used to develop these algorithms, and the algorithms could be updated by these companies at any time unbeknownst to the user [13, 14]. Thus, estimates of physical activity collected from the same device across time may provide different estimates of activity due solely to changes in the underlying algorithms that produce these metrics [13, 14].

An alternative, device-agnostic or monitor-independent approach may address these limitations by enabling data from any device to be processed using the same algorithm or processing methodology [15, 16]. A device-agnostic approach is a realistic possibility as consumer wearables have released application programming interfaces (API) that allow access to the raw accelerometry data (i.e., x, y, z axis readings collected by these devices) [17]. This has the potential to increase the comparability of physical activity estimates across time and between different consumer wearables and research-grade devices.

A necessary first step to applying a device-agnostic approach to raw accelerometry data collected by consumer wearables is to conduct mechanical signal testing of the data via controlled protocols [18]. This testing allows for the evaluation of device signals and their response to known stimuli. It also allows for the evaluation of reliability and validity of the raw acceleration output from consumer wearables without the influence of human variation [18]. It is also useful to evaluate the raw acceleration output from research-grade devices herein because it allows us to compare the acceleration output from research-grade and consumer wearables on the same metric, when compared to more direct estimates of acceleration from a mechanical shaker table. Therefore, this study will evaluate the between-device reliability and validity of the raw acceleration output from research-grade and consumer wearable devices, compared to accelerations produced by a mechanical shaker table as the reference metric. While studies have previously examined research-grade accelerometers with this methodology [19, 20], this is among the first studies to report shaker table outcomes evaluating the raw accelerometry data from consumer-grade devices.

## Methods

Raw accelerometry data from a total of 40 devices were evaluated in this study. The research-grade devices included n = 10 ActiGraph wGT3X-BT (ActiGraph; ActiGraph LLC Pensacola, FL). The consumer wearable devices included n = 10 Apple Watch Series 7 (Apple; Apple Technology Company, Cupertino, CA), n = 10 Garmin Viovactive 4S (Garmin; Garmin Ltd., Olathe, KS), and n = 10 Fitbit Sense (Fitbit; Google LLC, San Francisco, CA). Inter-device reliability and validity of raw accelerations for all devices were tested, with accelerations produced by a mechanical shaker table (Scientific Industries, Bohemia, NY; Mini-300 Orbital-Genie, Model 1500) as the reference. Each device was securely mounted directly to the twin ratcheting clamps of a mechanical shaker table (S1 Fig) that produces controlled oscillations at frequencies between approximately *f_shaker_* = 0.6 and 5 Hertz (Hz). We converted *f_shaker_* in Hz to acceleration using the expression for centripetal acceleration, *a_orbital_* = *v*^2^/*r_orbital_* [21], where *r_orbital_* is the radius of rotation for the orbital shaker *r_orbital_*. From the manual for this particular shaker (supplementary https://cdn.shopify.com/s/files/1/0489/6990/8374/files/SI-M1600_Manual.pdf?v=1617998279), the specified diameter of the orbit is 2*r_orbital_* = 1.9cm and the rotational speed is given by *v* = 2*πr_orbital_f_shaker_*, since for each complete cycle of 2*π* radians, the table traverses a distance of circumference 2*πr_orbital_* in time 1/*f_shaker_*. In other words:

$$a_{orbital}(cm/s^{2})=4\pi^{2}r_{orbital}f_{shaker}^{2}$$

to convert this acceleration to units of earth’s gravity (g’s), divide *a_orbital_* by 9.81cm/s^2^.

A total of five devices were placed on the shaker table at once. Serial number/device ID and position of devices (numbered 1 to 5 from left to right) were recorded for all devices. Prior to each trial, the shaker table was placed on a level surface (i.e., floor); time from each device was recorded at the second level.

### Device software

ActiGraphs were initialized to provide output from each directional axis using ActiLife software (version 6.13.4; ActiGraph LLC, Pensacola, FL). Garmin devices were initialized, and data were recorded in RawLogger (version 1.0.20211201a) and exported through Garmin Connect software^TM^. Apple devices were initialized, and data were recorded in SensorLog (version 5.2) and exported into comma-separated values (CSV) files via Health Auto Export (version 6.3). RawLogger and SensorLog are user-written apps that leverage the device-specific Application Programming Interface (API) to collect the underlying sensor data on the respective devices. RawLogger is available for download through the Connect IQ^TM^ store on the Garmin Connect^TM^ app, and SensorLog and Health Auto Export are available for download through the App Store. The research team developed a custom Fitbit app (Slog) leveraging the Fitbit API for the same purpose, and Fitbit devices were initialized, and data were recorded and exported through this app. The GitHub code for the custom Fitbit app is available athttps://github.com/ACOI-UofSC/Slog_HR. Sampling frequencies from 25 Hz to 100 Hz were recorded based on the capabilities of the ActiGraph (100 Hz), Apple (100 Hz), Garmin (25 Hz), and Fitbit (50 Hz).

### Reliability testing

Reliability testing included five identical devices mounted side-by-side (e.g., 5 ActiGraph devices) positioned 1–5 from left to right. Each device was tested for a total of 10 trials (5 trials at 0.6 Hz and 3.2 Hz) that lasted 2 minutes each [20]. A 15-second rest period took place at the beginning and end of each trial. Thus, it took ten minutes and 30 seconds to test 5 devices at one speed. The time of the 15-second rest periods and the trial start and end time were recorded based on device time. A minimum of 20 trials were conducted for each device brand, totaling 80 trials. Trials with missing data due to device malfunction: Apple (n = 20) and Fitbit (n = 10) were repeated to ensure that raw acceleration data from all devices could be analyzed. No trials had to be repeated for ActiGraph and Garmin devices.

### Validity testing

For validity testing, five identical devices were mounted side-by-side until all devices were run through the validity trials. The trials lasted 14 minutes and 30 seconds. Consistent with past validation studies [20, 22], each trial began with a 15-second rest period (i.e., no movement) followed by a standardized series of oscillations at seven frequencies (i.e., 3.2 Hz, 2.8 Hz, 2.4 Hz, 1.9 Hz, 1.5 Hz, 1.0 Hz, 0.6 Hz) lasting two minutes each. These frequencies were chosen because they are consistent with human movement ranging from 1.5 to 16 mph [23]. The start and stop times were noted at each frequency for both research-grade and consumer wearable devices. Each trial ended with another 15-second rest period. A minimum of 2 trials were conducted for each device brand, totaling 8 trials. Trials/devices with missing data due to device malfunction: Apple (n = 4) and Fitbit (n = 1) or shaker table malfunction (n = 1) were repeated to minimize missing data; no trials had to be repeated for ActiGraph or Garmin devices. Following all testing, raw acceleration data for both research-grade and consumer wearable devices were downloaded and converted to a CSV file using ActiLife software and the device-specific user-written apps, respectively.

### Sample size considerations

A sample size of 10 was selected to be consistent with previous research [19] and to provide reasonable variability within and between devices. Further, by selecting 10 devices the study was adequately powered to detect equivalence bounds of ±10% from the shaker table speed. Power is determined for an equivalence test by identifying the likelihood that the difference between two estimates is within prespecified equivalence bounds [24]. Power is then determined based upon the smallest acceptable width of the equivalence bounds. Power was calculated to detect equivalence between devices for estimates of light activity and MVPA. With a sample of 10 of each accelerometer, assuming an alpha of 0.05, and a standard deviation of the difference 10%, the study was adequately powered (power = 0.8) to detect equivalence bounds from -10% to 10% difference using standard statistical tests.

### Data processing

Raw acceleration data from all devices (i.e., ActiGraph, Apple, Garmin, and Fitbit) were extracted from the middle minute of each 2-minute oscillation frequency. Consistent with past research, Euclidean Norm Minus One (ENMO) was calculated [25–28]. All values were multiplied by 1000 (milligravity-mg) to be consistent with published intensity thresholds based on the GGIR package for accelerometry in R statistical software [29]. Data were aggregated to the second level by extracting the mean and root mean square (RMS) value for each second for all devices for ENMO. We calculated both mean and RMS, as both have been calculated previously, suggesting that there is no consensus on aggregation methods for raw accelerometry data [20, 22, 30].

### Correlation coefficients

Two-way random effects absolute intraclass correlation coefficients (ICC) were calculated to assess reliability for all devices. ICC values less than 0.50 were defined as poor reliability, between 0.50 and 0.75 as moderate reliability, between 0.75 and 0.90 as good reliability, and greater than 0.90 as excellent reliability [31]. Prior to statistical analyses for validity testing, descriptive means and standard deviations for the mean and RMS were calculated across devices for each speed ranging from 0.6 to 3.2 Hz. For the validity testing, Pearson product moment *(r)* and Lin’s concordance correlation coefficient (CCC) were calculated to assess correlation and agreement of raw acceleration data from ActiGraph and consumer wearable devices compared to the reference (i.e., acceleration from the shaker table) [32]. Pearson product moment interpretations were defined based on Dancey and Reidy [33], and Lin’s concordance correlation coefficient was defined similarly based on recommendations from Altman (1991), with coefficients less than 0.20 as poor and greater than 0.80 as excellent [34].

### Discrepancy analyses

An absolute error was calculated to assess the magnitude of the error between the reference metric and the raw acceleration data from ActiGraph and consumer wearable devices. The mean bias was also calculated to assess whether the raw acceleration output from ActiGraph and consumer wearable devices over- or underestimated acceleration output compared to the reference metric. Raw acceleration data from one ActiGraph (ID = 210) was eliminated because the device was faulty and provided implausible acceleration values (all ENMO values were below 0). Thus, there were (N = 3,780) observations for ActiGraph, whereas Apple and Garmin devices contributed (N = 4,200) observations. Missing data were present across all Fitbit devices except two, which contributed to (N = 3,975) observations for Fitbit.

### Equivalence testing

Following the discrepancy analyses above, the Two-One-Sided-Tests method [35] was adopted to assess the equivalence of the raw accelerometry data collected from the accelerometers compared with accelerations from the shaker table [36]. For equivalence testing, the null hypothesis is that the raw data collected via the accelerometers and the shaker table speeds are not equivalent. To test this 90% equivalence bounds are required [37]. An equivalence zone of ±10% was adopted based upon previous work and industry standards [37, 38]. Thus, should the 90% confidence interval of the accelerometer data fall completely within ±10% of the shaker table speed, equivalence is concluded. The ‘tost’ command in Stata was used to complete all equivalence analyses.

## Results

For reliability, ICCs (95% confidence intervals) are presented for the raw acceleration data from all devices for both aggregation methods (i.e., mean and RMS) for all devices in Table 1. The ICCs for ActiGraph were 0.97 (0.92, 0.99) and 0.97 (0.93, 0.98) for the mean and RMS aggregation methods, respectively. The ICCs for Apple were 0.99 (0.99, 0.99) and 0.99 (0.99, 1.00) for the mean and RMS, respectively. The ICCs for Garmin were 0.88 (0.82, 0.92) and 0.90 (0.85, 0.93) for the mean and RMS aggregation methods, respectively. The ICCs for Fitbit were 0.88 (0.86, 0.89) and 0.87 (0.85, 0.88) for the mean and RMS aggregation methods, respectively.

**Table 1:** Summary of intraclass correlation coefficients for all devices aggregated based on the mean and root mean square.

Device	Mean	95CI	RMS	95CI
ActiGraph	0.97	(0.92, 0.99)	0.97	(0.93, 0.98)
Apple	0.99	(0.99, 0.99)	0.99	(0.99, 1.00)
Garmin	0.88	(0.82, 0.92)	0.90	(0.85, 0.93)
Fitbit	0.88	(0.86, 0.89)	0.87	(0.85, 0.88)

Abbreviations: “95CI” 95% confidence interval, “RMS” root mean square

**Fig 1:**
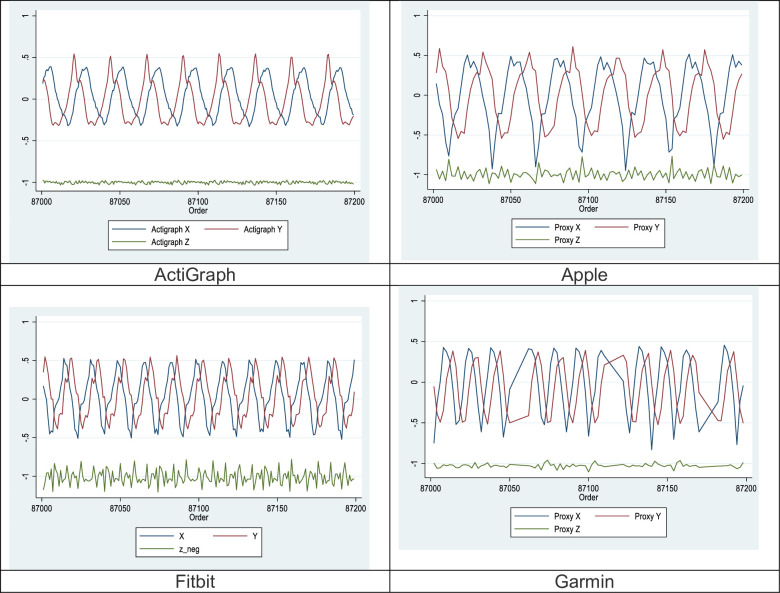
Raw signals with baselines for all four monitors at 1.9 Hz.

**Fig 2:**
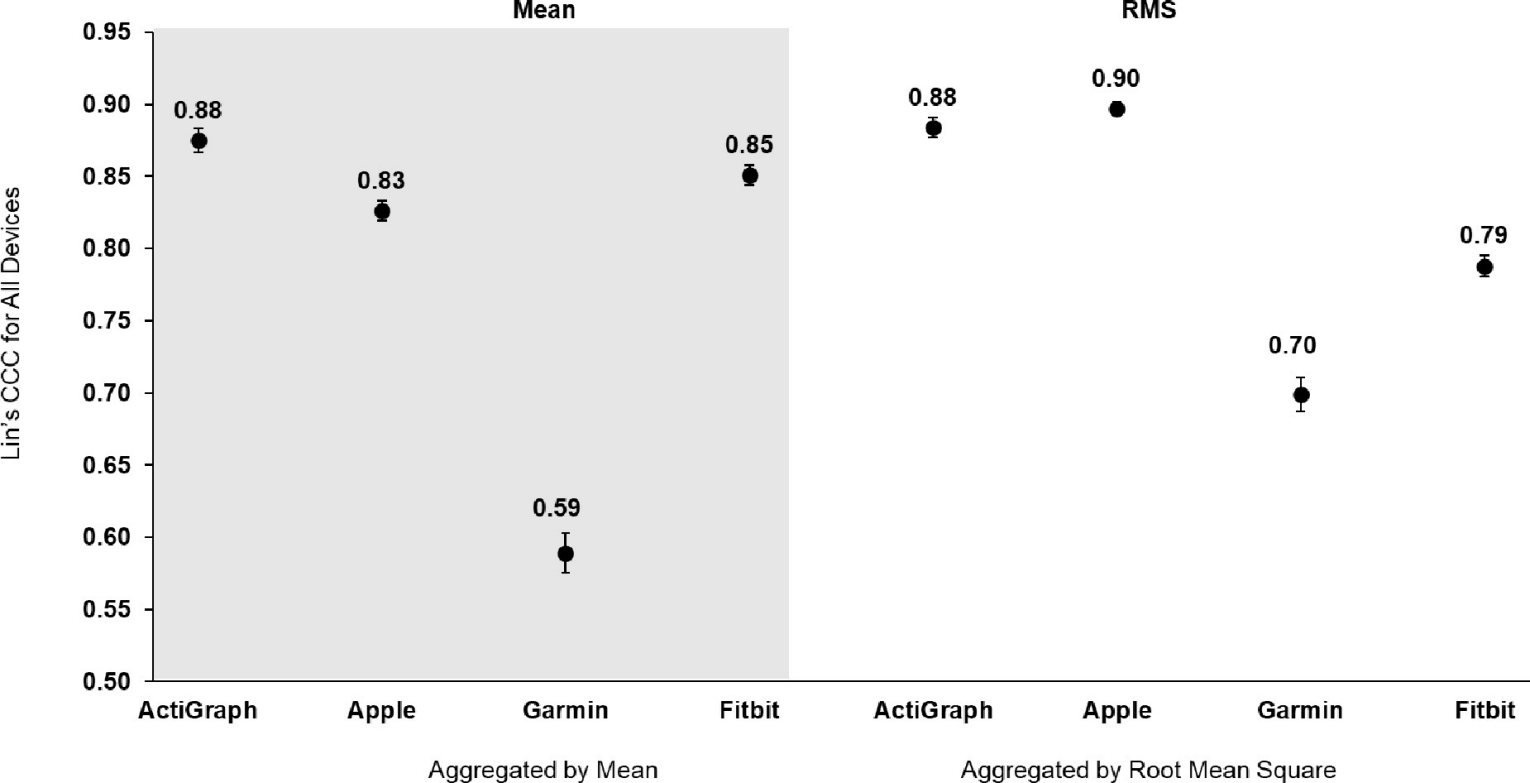
Lin’s concordance correlation coefficient of the raw acceleration data from all devices compared to the accelerations produced by a mechanical shaker table. Error bars represent standard error.

**Fig 3:**
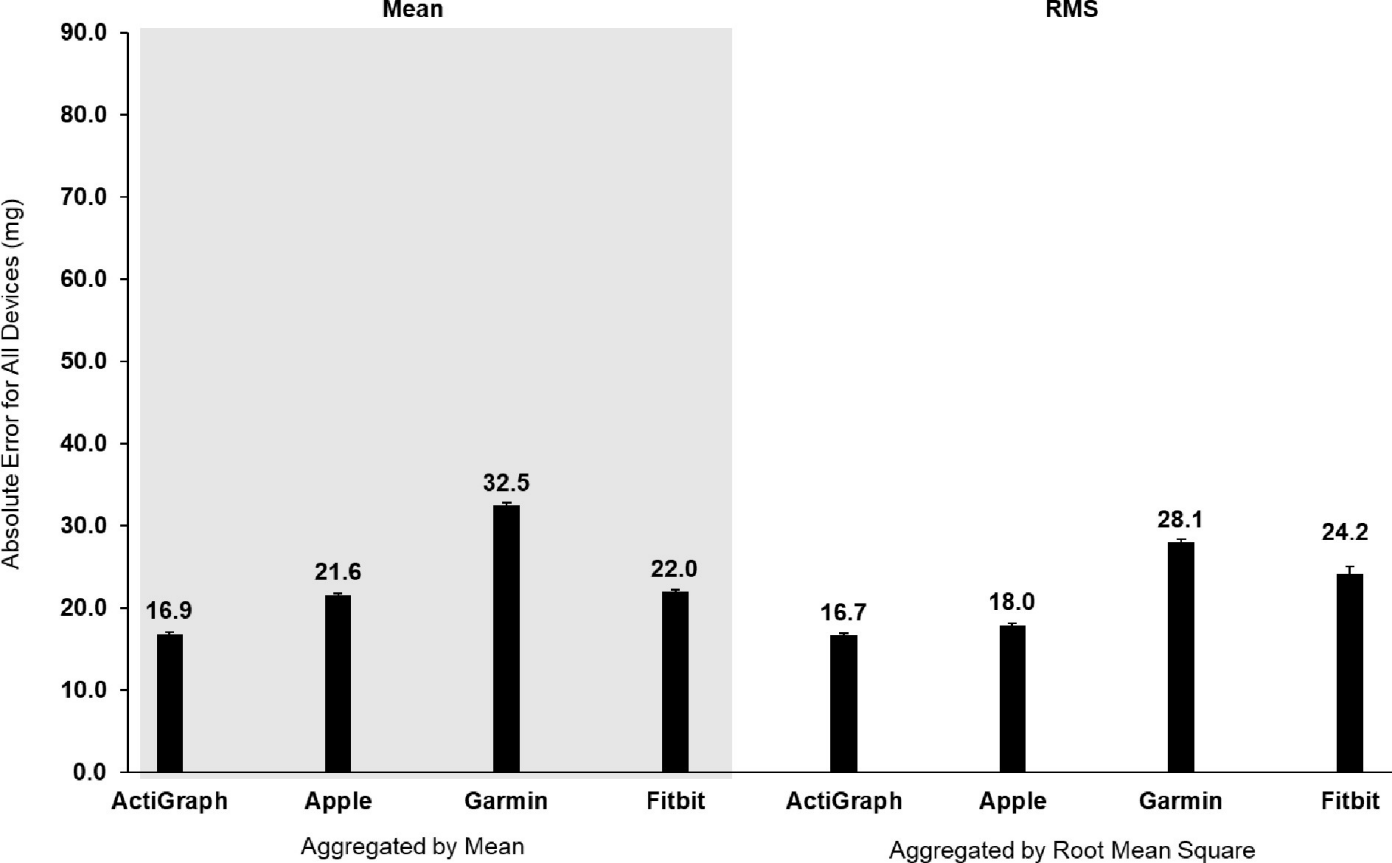
Absolute error of the raw acceleration data from all devices compared to the accelerations produced by a mechanical shaker table. Error bars represent standard error.

**Fig 4:**
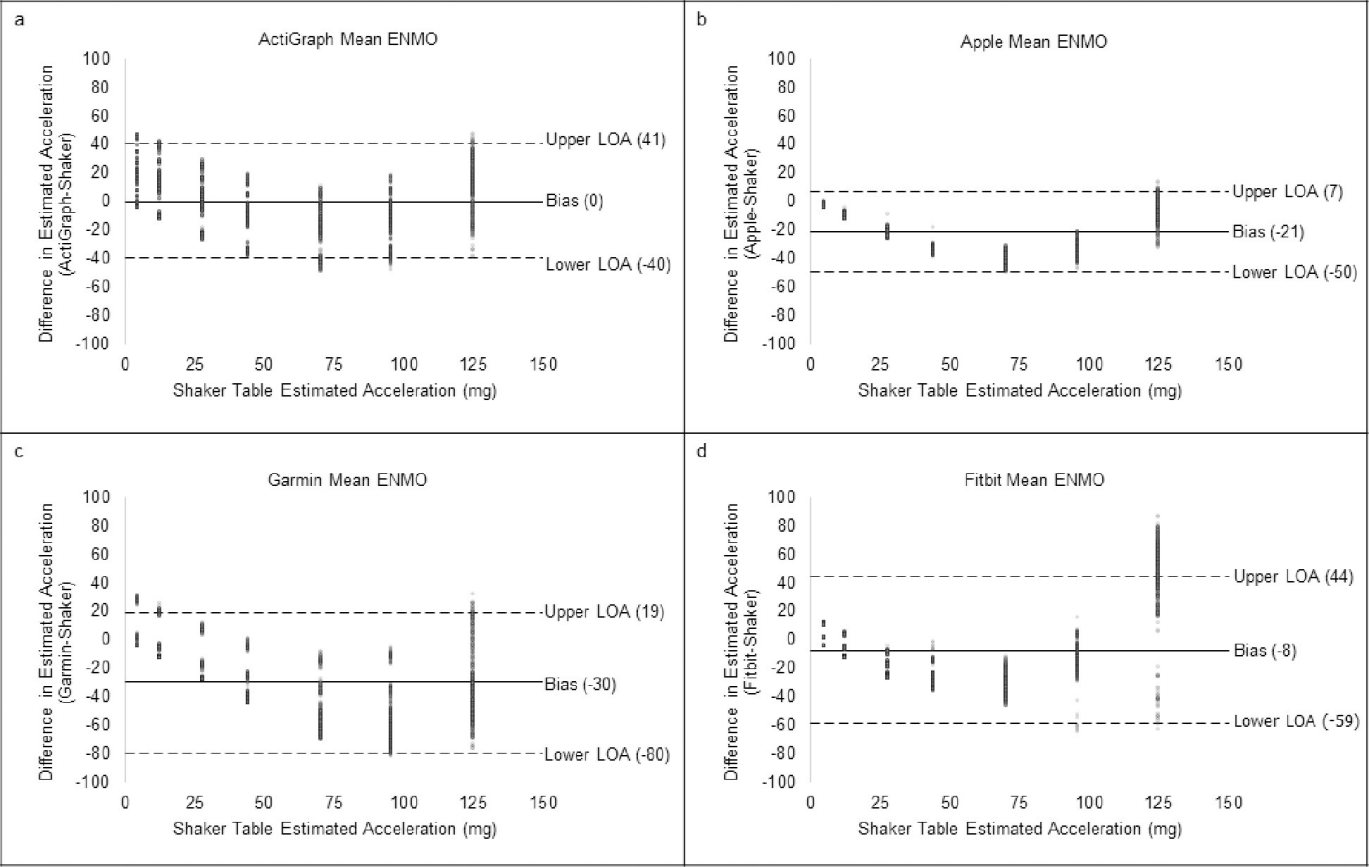
Bland-Altman plots of estimated mean ENMO from all devices compared to estimated shaker table acceleration.

**Fig 5:**
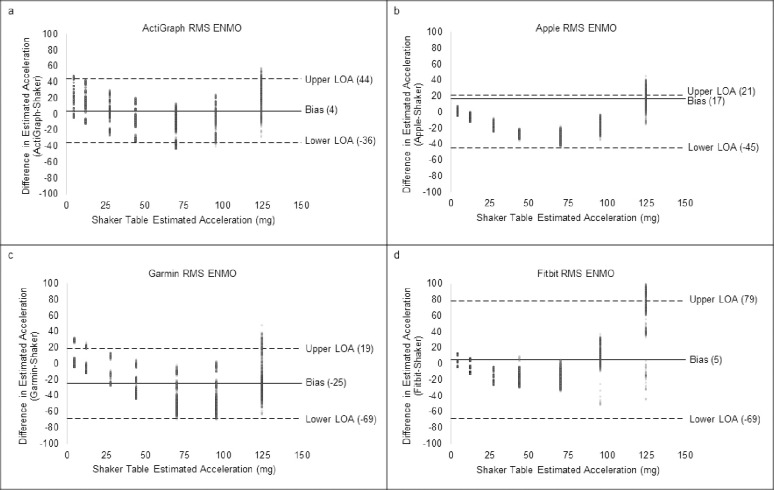
Bland-Altman plots of estimated root mean square ENMO from all devices compared to estimated shaker table acceleration.

For validity, a summary table of outcomes based on the raw acceleration data from all devices is presented in Table 2. Fig 1 shows the raw signals with baselines for all four monitors at 1.9 Hz. Fig 2 shows the concordance of the raw acceleration data from all devices compared to the reference metric. Fig 3 shows the absolute error of the raw acceleration data from all devices compared to the reference metric. Fig 4 are Bland-Altman plots based on the estimated mean ENMO for each device compared to accelerations from the reference metric. Fig 5 are Bland-Altman plots based on the estimated RMS ENMO for each device compared to accelerations from the reference metric.

**Table 2:** Summary statistics for all devices based on the mean and root mean square aggregation methods.

	Devices	ActiGraph	Apple	Garmin	Fitbit
**Mean**	Observations	3,780	4,200	4,200	3,975
	Mean (mg)	54.4	32.7	23.8	46.1
	SD (mg)	41.5	41.0	34.1	57.4
	Pearson’s r	0.88	0.94	0.79	0.91
**Root Mean Square**	Observations	3,780	4,200	4,200	3,975
	Mean (mg)	58.1	41.8	29.0	58.8
	SD (mg)	45.0	48.9	37.9	71.8
	Pearson’s r	0.89	0.94	0.84	0.92

Abbreviations: “SD” standard deviation, “mg” = milligravity

Pearson product moment correlations between raw accelerometry estimates for ActiGraph and the reference metric were *r =* 0.88 and *r =* 0.89 for the mean and RMS aggregation methods, respectively. CCCs (95% confidence intervals) when compared to the shaker table were *r*_*c*_ = 0.88 (0.87, 0.88) and *r*_*c*_ = 0.88 (0.88, 0.89) for the mean and RMS aggregation methods, respectively. Mean bias (95% confidence intervals) was 0.0mg (-40.0, 41.0) and 4.0mg (-36.0, 44.0), and absolute error was 16.9mg and 16.7mg for the mean and RMS aggregation methods, respectively.

Pearson product moment correlations between raw accelerometry estimates for Apple and the reference metric were *r =* 0.94 and *r =* 0.94 for the mean and RMS aggregation methods, respectively. CCCs when compared to the shaker table were *r*_*c*_ = 0.83 (0.82, 0.83) and *r*_*c*_ = 0.90 (0.89, 0.90) for the mean and RMS aggregation methods, respectively. Mean bias (95% confidence intervals) was -21.0mg (-50.0, 7.0) and -12.0mg (-45.0, 21.0), and absolute error was 21.6mg and 18.0mg for the mean and RMS aggregation methods, respectively.

Pearson product moment correlations between raw accelerometry estimates for Garmin and the reference metric were *r =* 0.79 and *r =* 0.84 for the mean and RMS aggregation methods, respectively. CCCs when compared to the shaker table were *r*_*c*_ = 0.59 (0.58, 0.60) and *r*_*c*_ = 0.70 (0.69, 0.71) for the mean and RMS aggregation methods, respectively. Mean bias (95% confidence intervals) was -30.0mg (-80.0, 19.0) and -25.0mg (-69.0, 19.0), and absolute error was 32.5mg and 28.1mg for the mean and RMS aggregation methods, respectively.

Pearson product moment correlations between raw accelerometry estimates for Fitbit and the reference metric were *r =* 0.91 and *r =* 0.92 for the mean and RMS aggregation methods, respectively. CCCs when compared to the shaker table were *r*_*c*_ = 0.85 (0.84, 0.86) and *r*_*c*_ = 0.79 (0.78, 0.80) for the mean and RMS aggregation methods, respectively. Mean bias (95% confidence intervals) was -8.0mg (-59.0, 44.0) and 5.0mg (-69.0, 79.0), and absolute error was 22.0mg and 24.2mg for the mean and RMS aggregation methods, respectively.

Findings from the equivalence tests between the raw acceleration estimates from all devices and the reference metric are presented in Table 3. No device estimates were found to be statistically significantly equivalent no matter the aggregation method when compared to the reference metric. For ActiGraph, mean differences were -12.9 and -9.1 based on the mean and RMS aggregation methods, respectively. For Apple, mean differences were -29.6 and -20.5 based on the mean and RMS aggregation methods, respectively. For Garmin, mean differences were -38.5 and -34.2 based on the mean and RMS aggregation methods, respectively. For Fitbit, the mean differences were -16.2 and -3.5 based on the mean and RMS aggregation methods, respectively.

**Table 3:** Equivalence testing for ActiGraph, Apple, Garmin, Fitbit.

		Mean Difference	Lower 90% Bound	Upper 90% Bound	Interpretation
Mean	ActiGraph	-12.9	-15.8	-10.1	not significantly within the equivalence bounds
	Apple	-29.6	-32.4	-26.8	not significantly within the equivalence bounds
	Garmin	-38.5	-40.8	-36.2	not significantly within the equivalence bounds
	Fitbit	-16.2	-20.0	-12.3	not significantly within the equivalence bounds
RMS	ActiGraph	-9.1	-12.1	-6.0	not significantly within the equivalence bounds
	Apple	-20.5	-23.8	-17.2	not significantly within the equivalence bounds
	Garmin	-34.2	-35.5	-33.0	not significantly within the equivalence bounds
	Fitbit	-3.5	-8.3	1.3	not significantly within the equivalence bounds

Equivalence was set at 10% of the shaker speed (6.3mg) and differences were required to be completely within (±) these bounds to be considered equivalent

Abbreviations: “RMS” Root Mean Square, “mg” milligravity

## Discussion

The aim of this study was to evaluate the between-device reliability and validity of the raw acceleration output from research-grade (i.e., ActiGraph wGT3X-BT) and consumer wearable devices (i.e., Apple Watch Series 7, Garmin Vivoactive 4S, and Fitbit Sense) compared to accelerations produced by a mechanical shaker table. The raw acceleration data collected from all devices exhibited good-to-excellent between-device reliability based on the mean and RMS aggregation methods. For validity, the raw acceleration data from all devices exhibited a strong positive correlation to the reference metric with moderate-to-excellent concordance no matter the aggregation method. Except for Garmin, the raw acceleration data collected from consumer wearables demonstrated absolute errors with the reference metric that were similar to ActiGraph. However, equivalence testing revealed raw accelerometry data from all devices were not significantly within the equivalence bounds of the shaker speed. Moreover, the raw acceleration data collected from consumer wearables underestimated acceleration output to a greater degree than ActiGraph, when compared to the accelerations produced by the mechanical shaker table. Overall, the raw acceleration data for all devices differed when data were aggregated based on the mean and RMS for each second, with values generally being more reliable and accurate based on the RMS aggregation method.

A key finding of this study is that the reliability of the raw accelerometry estimates for Apple, Garmin, and Fitbit were similar to ActiGraph. In fact, consumer wearables exhibited moderate-to-excellent ICC values, with Apple demonstrating nearly perfect reliability (ICC of 0.99). These findings are similar to other studies evaluating the between-device reliability of research-grade devices using a mechanical shaker table. For instance, Powell et al. [39] reported an ICC of 0.99 between 23 RT3 accelerometers and Santos-Lozano et al. [19] reported an ICC of 0.97 between 10 ActiGraph GT3X accelerometers. More recently, studies have explored within-device reliability of various accelerometers and have reported ICCs ranging from 0.77 to 1.00 [40, 41]. Thus, ICCs presented in this study suggest that raw acceleration data collected from Apple, Garmin, and Fitbit provide reliable estimates of movement.

In the present study, it is also important to note that raw accelerometry estimates collected from Apple and Fitbit exhibited correlation and concordance with the reference metric that was consistent with ActiGraph. On the other hand, raw acceleration data collected from Garmin exhibited less correlation and concordance with the reference metric than ActiGraph. Our findings for Apple and Fitbit correlation are more consistent with a previous study that reported an excellent Pearson correlation (*r* = 0.97) between accelerations produced by GENEA accelerometers and a mechanical shaker table [30]. These findings suggest that raw acceleration data from Apple and Fitbit produce estimates of movement that are similar to raw acceleration data from ActiGraph. However, more information is needed to determine if the raw acceleration data from Garmin can be used to accurately estimate movement. These findings could be due to hardware differences between devices. For example, the dynamic accelerometer range of the ActiGraph is ±8g [42], while the default accelerometer range for Fitbit is ±4g [43]. The dynamic accelerometer range is an estimate of the greatest amount of acceleration that a device can accurately assess, and thus the relatively smaller accelerometer range of Garmin and Fitbit compared to ActiGraph could have led to more error in Garmin and Fitbit estimates at greater frequencies (S2 and S3 Figs). Differences in the raw acceleration output collected from ActiGraph and the consumer wearables could also be due to the post-processing of the raw data, which has been described previously [20].

Further evidence revealed that, compared to the reference metric, raw acceleration estimates from Apple and Fitbit exhibited absolute differences that were similar to the raw acceleration estimates from ActiGraph. On the other hand, raw acceleration estimates from Garmin exhibited larger absolute errors relative to the raw acceleration estimates from ActiGraph. It is also important to note that raw acceleration data from Apple and Garmin underestimated acceleration output by more than 20mg and 30mg, respectively, compared to raw acceleration estimates from ActiGraph. This is concerning for Garmin, since published intensity thresholds derived from ActiGraph data worn on the non-dominant wrist indicate that sedentary thresholds for children (7-11yrs) are under 35.6mg [26, 27]. Based on these intensity thresholds, it would be difficult to distinguish between sedentary and light intensity thresholds for children using raw acceleration output from Garmin. This may suggest that we need to move away from cut-points, especially since a device-agnostic approach may allow for increased comparability of physical activity estimates across time and between consumer wearables and research-grade devices. One way to summarize raw acceleration data in a device-agnostic manner is to generate open-source Monitor-Independent Movement Summary units (MIMS-units) [44]. MIMS-units could increase the standardization of raw data processing from different devices and reduce between-device variability in estimates of movement [44].

Overall, the findings suggest that raw acceleration output from Apple and Fitbit are similar to raw acceleration output from ActiGraph. However, no device estimates were found to be statistically significantly equivalent to accelerations produced by the reference metric. These limitations with accelerometry are well-documented for distinguishing between sedentary and light activity. For instance, a study using 2-regression models to estimate energy expenditure derived from ActiGraph counts in children (7-13yrs) observed mean absolute percent error values that ranged from 32.5% to 39.4% and 14.5% to 42.9% for sedentary and light activities, respectively [45]. A similar study reported that research-grade accelerometers (i.e., ActiGraph, Actical, and AMP-331) tended to overestimate sedentary and light activities in adults [46]. Though most of the evidence on the associations of device-based sedentary behavior and health is based on accelerometers that infer sedentary time from a lack of movement, this can lead to misclassification of low-movement, non-sedentary behaviors as sedentary behaviors [47]. The absolute errors of ActiGraph, Apple, and Fitbit (~20mg) compared to the reference metric suggest that the relatively small window for sedentary behavior (under 35.6mg) may pose an issue for estimating physical activity outcomes from accelerometry [29]. Therefore, additional metrics (i.e., heart rate) may need to be combined with accelerometry to improve estimates of these outcomes. An advantage of consumer wearables is their ability to collect accelerometry and heart rate data simultaneously. Thus, it may be possible to leverage the raw acceleration and heart rate data from consumer wearables (i.e., Apple and Fitbit) to overcome limitations with accelerometry alone for estimating physical activity outcomes.

There were several strengths of the present study. The first strength is that accelerations produced by a mechanical shaker table served as the reference to assess the reliability and validity of accelerations produced by various accelerometers. This method allowed for a highly controlled, repeatable evaluation of underlying accelerations produced by various accelerometers shaken in orbital motion at known frequencies. Another strength is that the raw accelerations from devices were evaluated, allowing for between-monitor comparisons of accelerations through elimination of proprietary signal processing that has traditionally been used to derive activity counts from research-grade devices [20]. Additionally, this study evaluated the raw accelerations from consumer wearables, addressing concerns about the proprietary signal processing of these devices [48]. By evaluating the raw accelerations for both research-grade and consumer wearable devices, we were able to compare estimates from the devices on the same metric (mg). Lastly, we calculated Lin’s CCC, absolute error, mean bias, and equivalence testing to assess the agreement of the raw accelerometry data from research-grade and consumer wearable devices compared to accelerations produced by a mechanical shaker table. This allowed us to evaluate the agreement of the accelerations between proxy and reference, the overall error of the raw acceleration estimates, and the direction of the average error of the estimates from all devices, whereas other studies only used Pearson correlation to assess validity [22, 30].

Pearson correlation merely measures the covariance between two variables, not the agreement or error. Using these statistics, we were also able to compare the validity metrics produced by the raw acceleration estimates from consumer wearables to the validity metrics produced by the raw acceleration estimates from a research-grade device. This provided preliminary evidence for using the raw acceleration output from consumer wearables to estimate physical activity outcomes. However, the raw acceleration output from consumer wearables needs to be evaluated in settings that resemble free-living activities for children.

The limitations of the present study also need to be acknowledged. The first limitation is that there may have been between trial variability in speed across trials that would systematically affect the findings herein. Another limitation may be the technological advances that have occurred in the consumer wearables evaluated during the project. For instance, the Apple Watch Series 8 was released during the project. However, most of the technological advancements between the Apple Watch Series 7 and the Apple Watch Series 8 are centered on the dual-core processor and the addition of a temperature sensor [49], and thus may not impact accelerometer estimates between devices. Yet, information about the hardware of accelerometers used in consumer wearable devices is largely proprietary. Another limitation may be the post-processing of the raw acceleration data for all devices [20]. The post-processing of the raw acceleration data for all devices is proprietary, so the data is not truly raw. It is also unclear why missing data were present across all Fitbit devices except two. This may have been due to software malfunction with the custom Fitbit app (Slog) that was used to leverage the Fitbit Application Programming Interface.

## Conclusions

Findings from this study suggest that raw accelerometry data from Apple, Garmin, and Fitbit are reliable and provide estimates of raw accelerometry that are similar to ActiGraph, except for Garmin. Additionally, no raw accelerometry estimates were statistically significantly equivalent to the reference. Thus, harmonization approaches across devices like MIMs may be necessary if a truly device-agnostic approach is to be adopted. Future studies should explore using device-agnostic and data harmonization approaches for estimating physical activity from raw accelerometry data produced by Apple and Fitbit in settings that resemble free-living activities for children.

## Supporting information

S1 Fig Orbital mechanical shaker used for testing. (DOCX)

S2 Fig Absolute error of the raw acceleration data from all devices by speed compared to the accelerations produced by a mechanical shaker table. Error bars represent standard error.
(DOCX)

S3 Fig Mean bias of the raw acceleration data from all devices by speed compared to the accelerations produced by a mechanical shaker table. Error bars represent standard error.
(DOCX)

S1 File Analyses collapsed data_v11. (XLSX)

S2 File Analyze data all devices STATA code. (TXT)

S1 Dataset Apple with shaker speeds_aggregated_v2 –dataset. (XLSX)

S2 Dataset Fitbit with shaker speeds_aggregated_v4 –dataset. (XLSX)

S3 Dataset Garmin with shaker_speeds_aggregated_v2 –dataset. (XLSX)

S4 Dataset Actigraph_aggregated_v3 drop Actigraphid210 –dataset. (XLSX)
