# Supplementary figures and images for: Comparison of raw accelerometry data from ActiGraph, Apple Watch, Garmin, and Fitbit using a mechanical shaker table
Source: PLoS One. 2024 Mar 29;19(3):e0286898. doi: 10.1371/journal.pone.0286898 (PMC10980217; doi:10.1371/journal.pone.0286898)

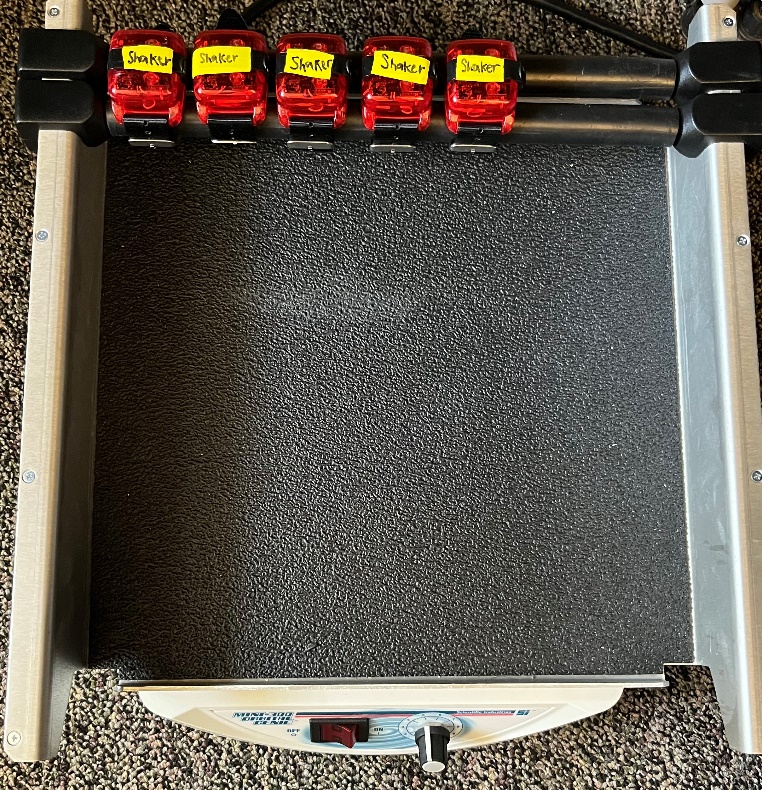

Supplement: S1 Fig — (DOCX) [file pone.0286898.s001.docx]

| a 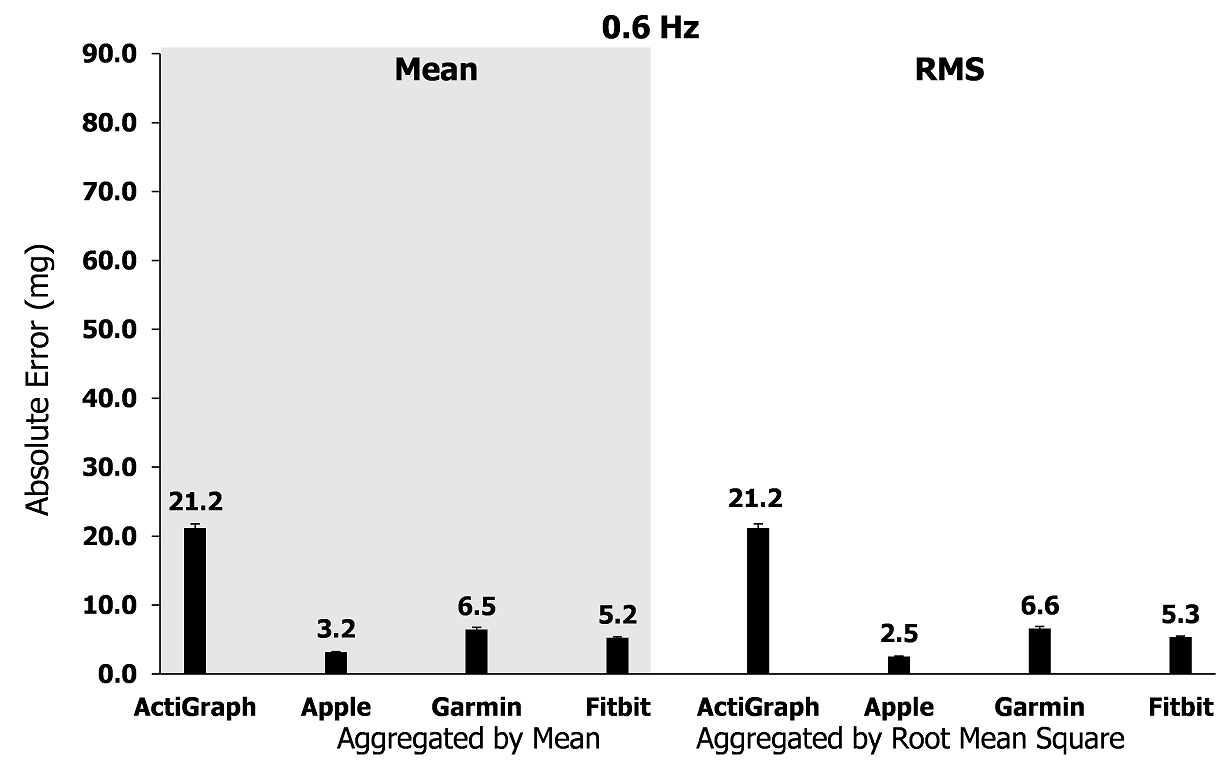 | b 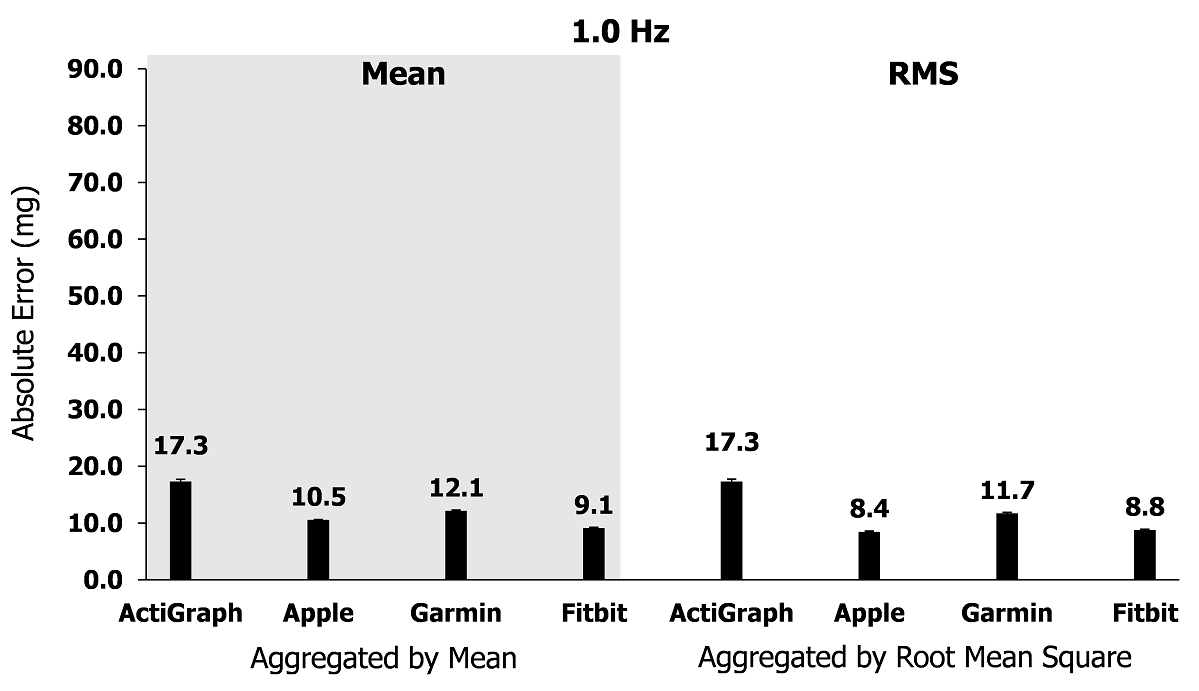 |
| --- | --- |
| c 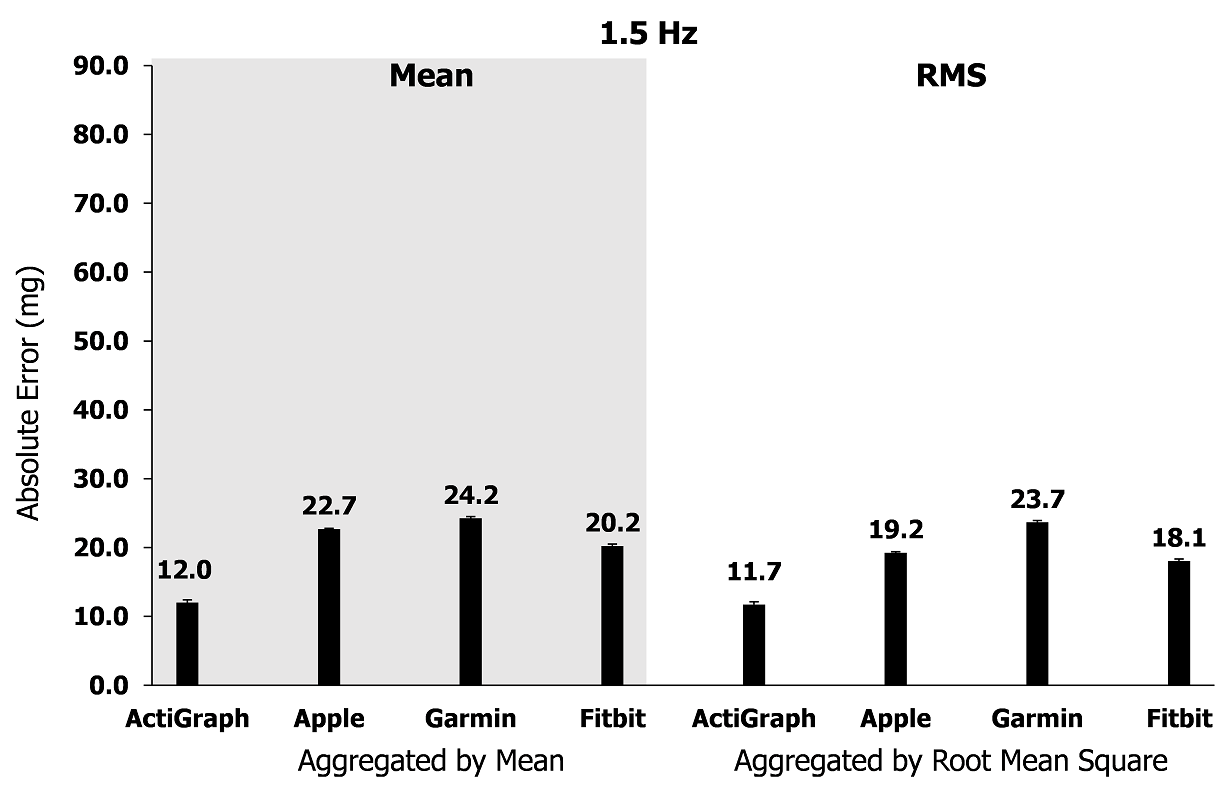 | d 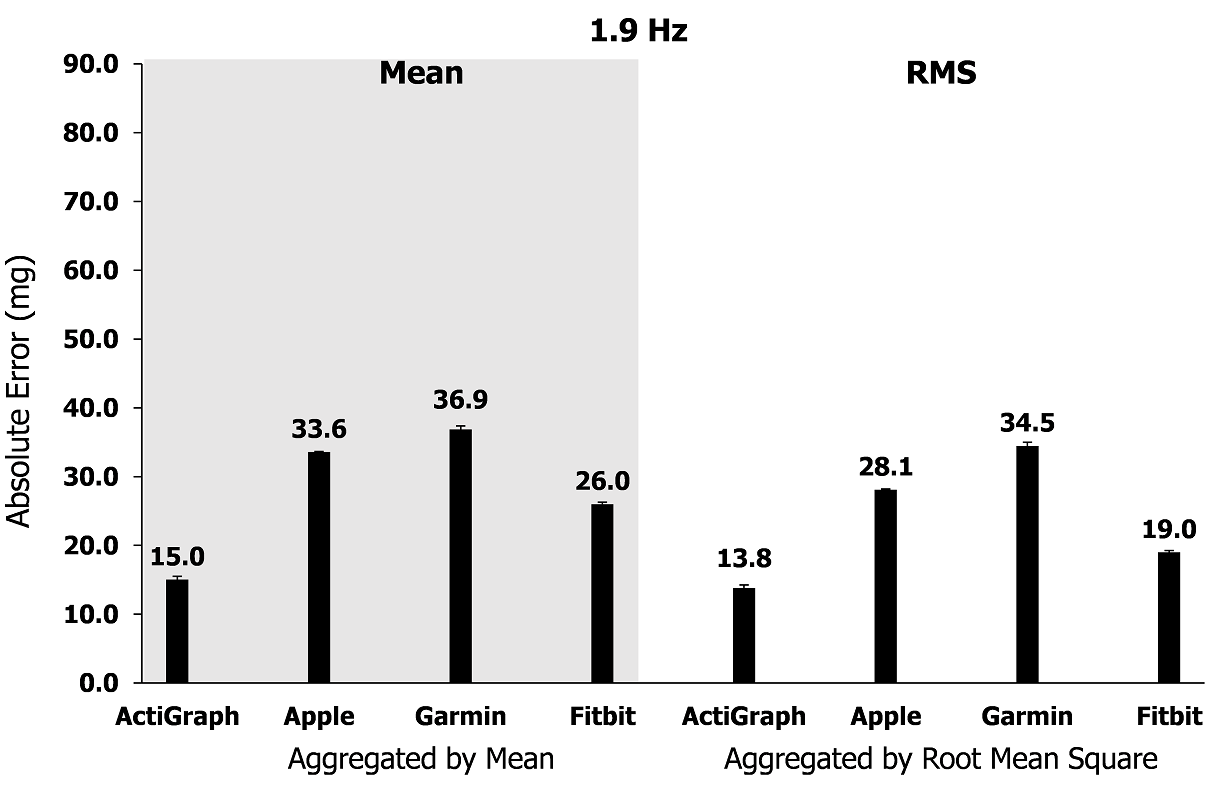 |
| e 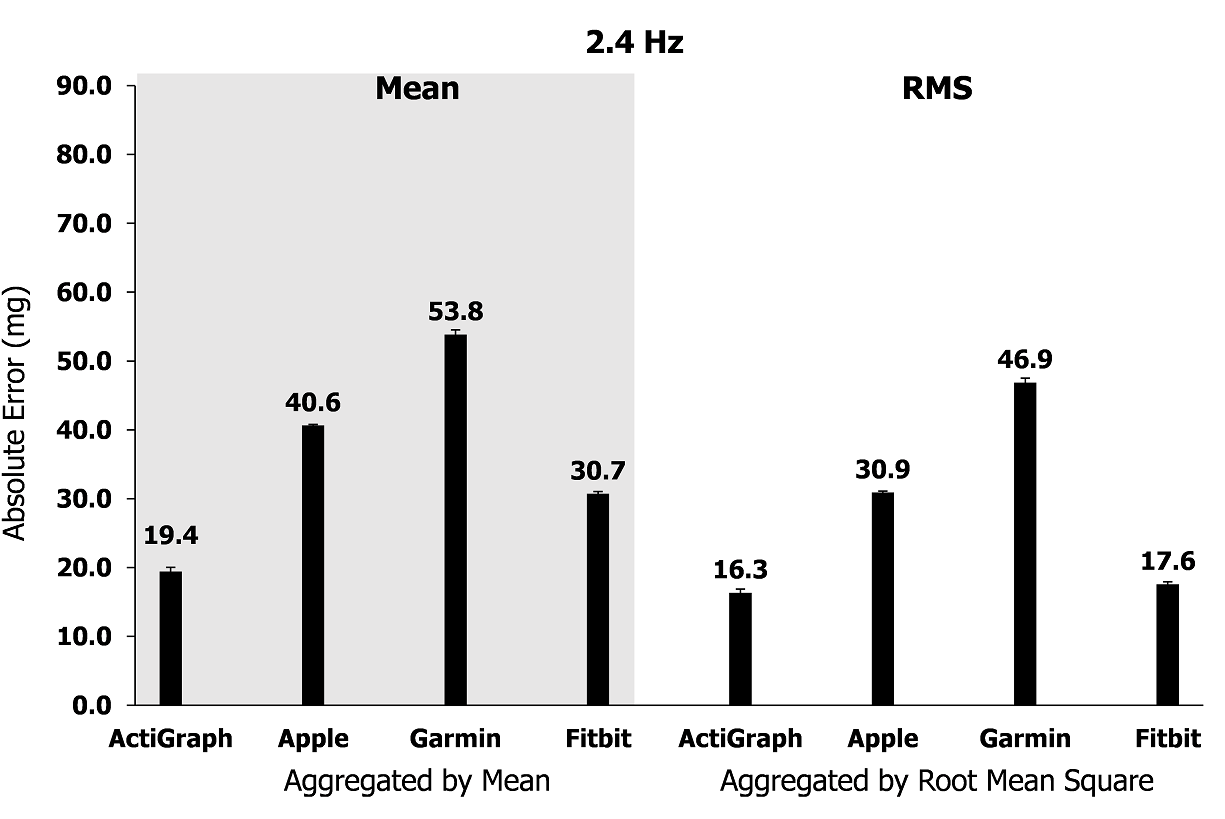 | f 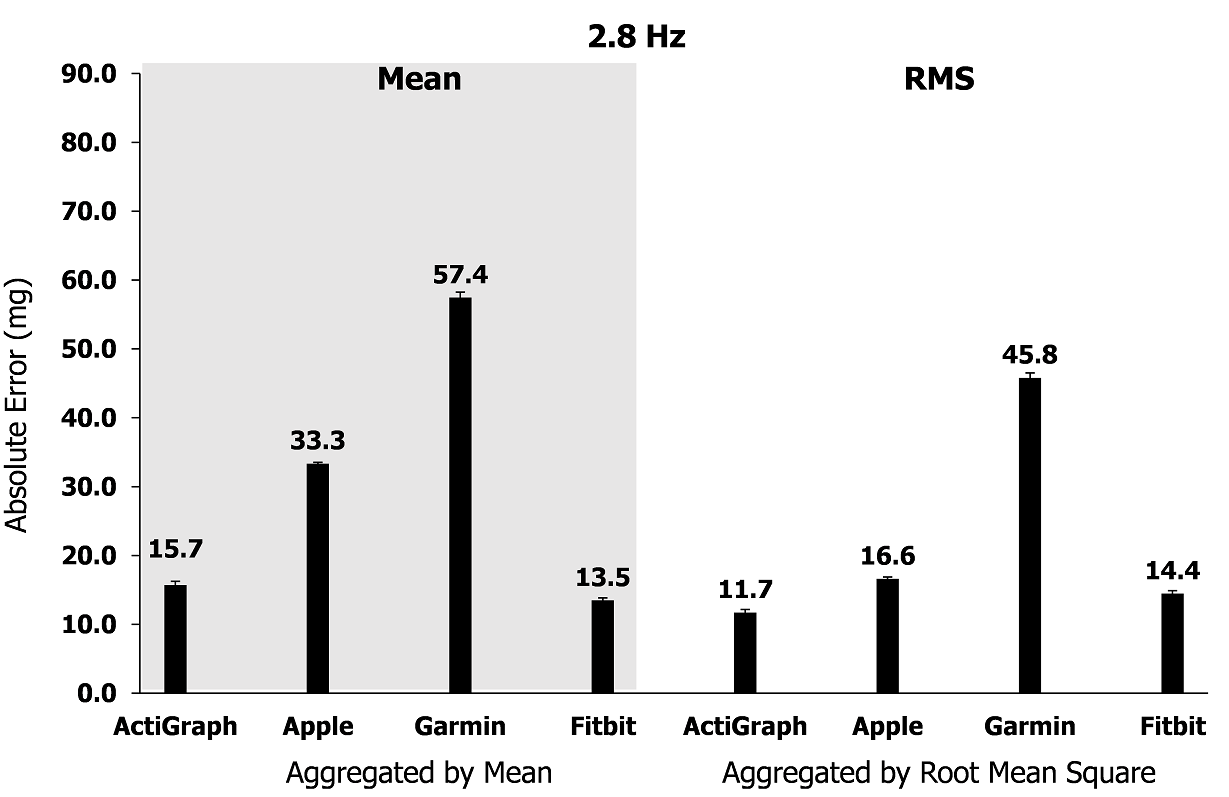 |
| g 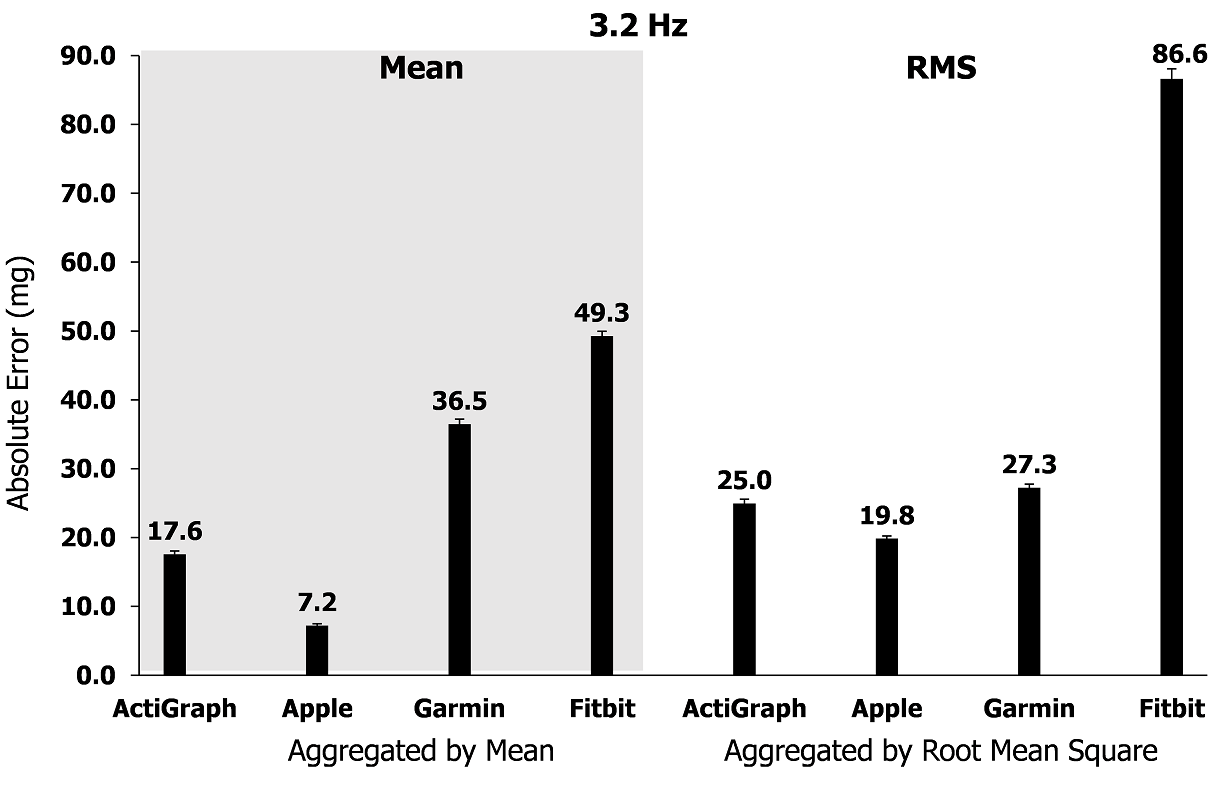 |

Supplement: S2 Fig — Error bars represent standard error. (DOCX) [file pone.0286898.s002.docx]

| a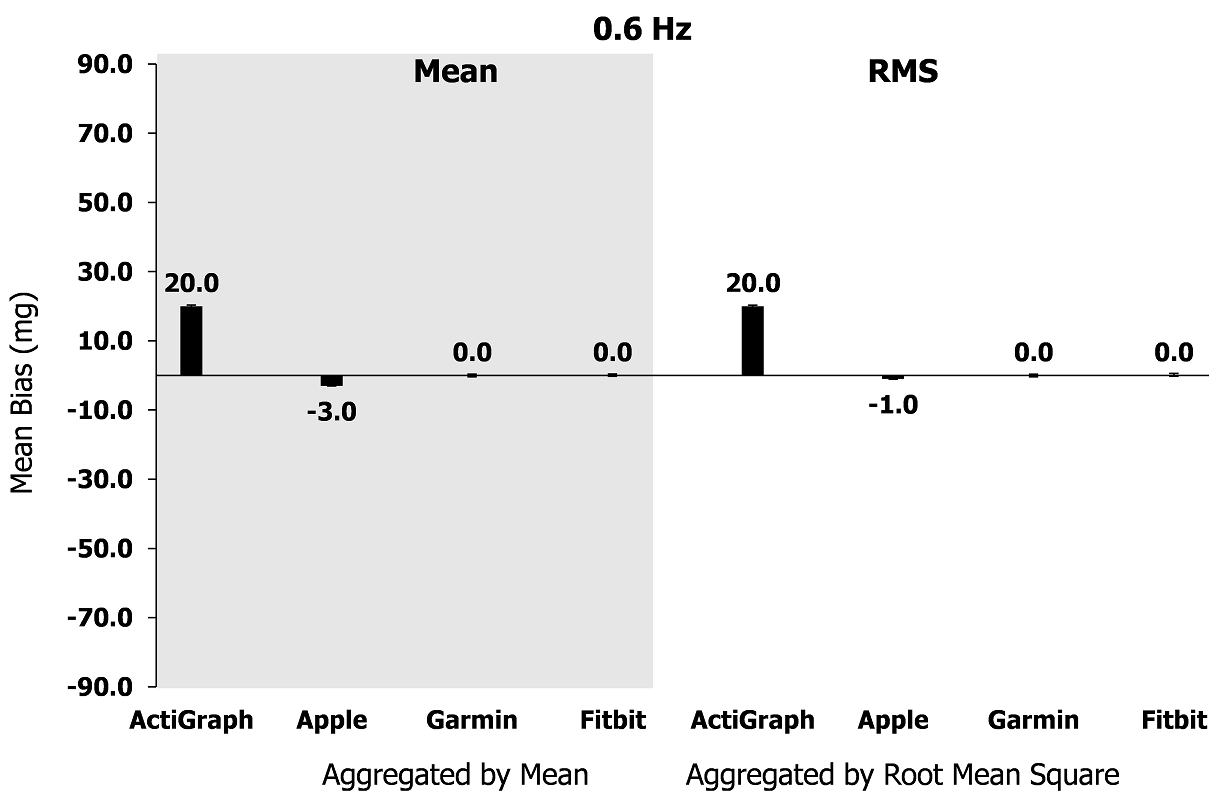 | b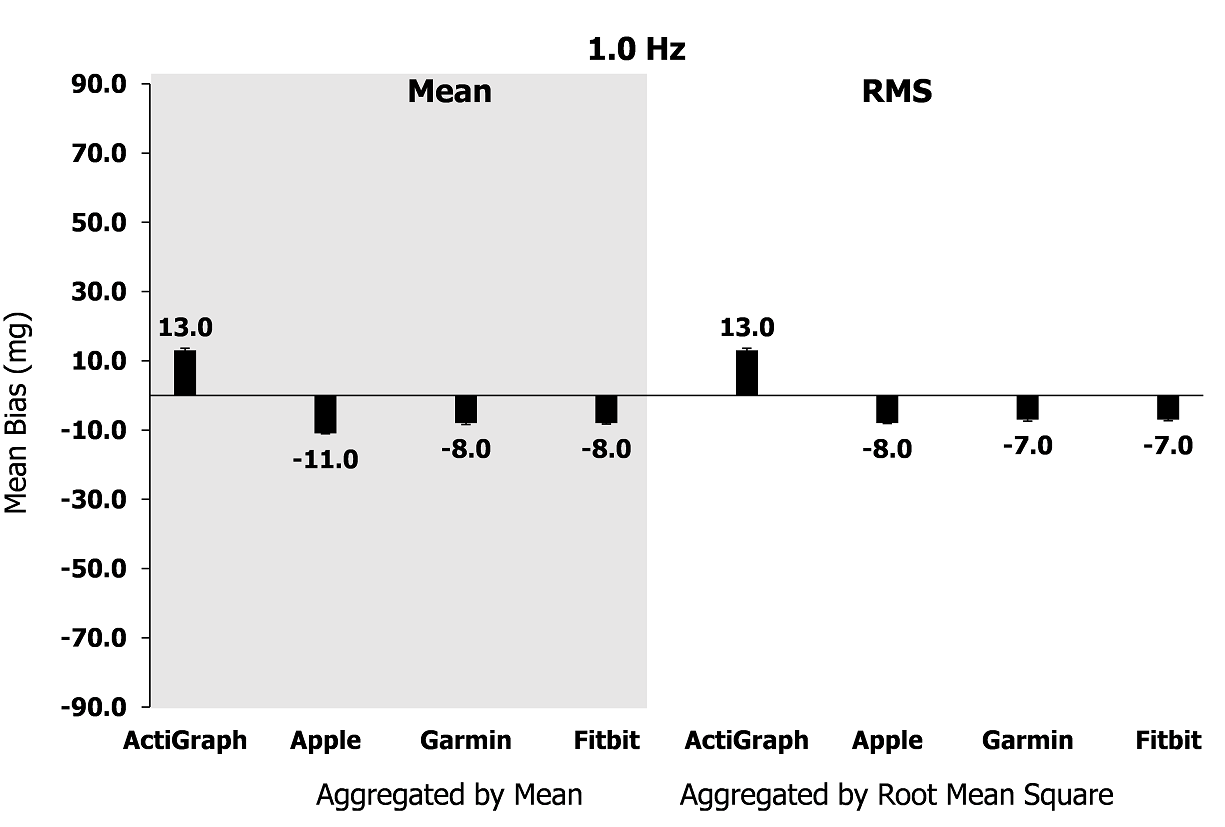 |
| --- | --- |
| c 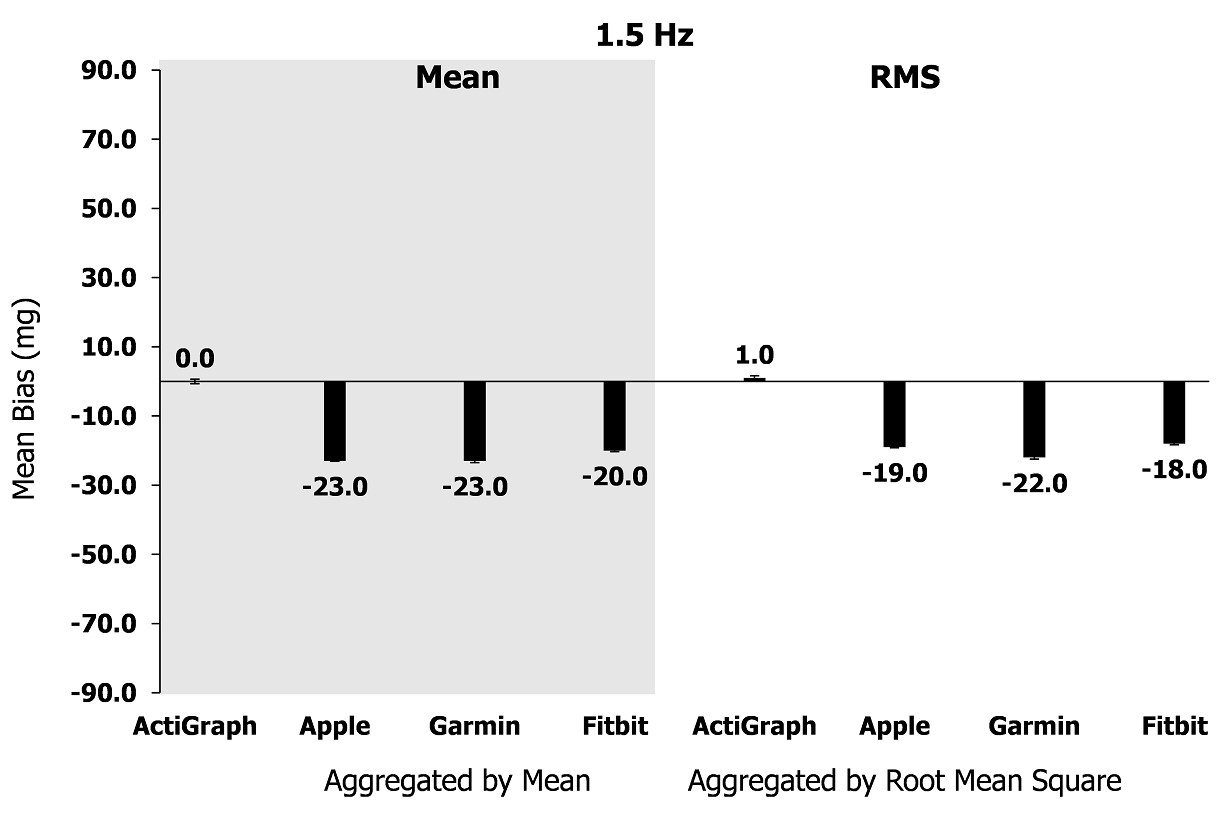 | d 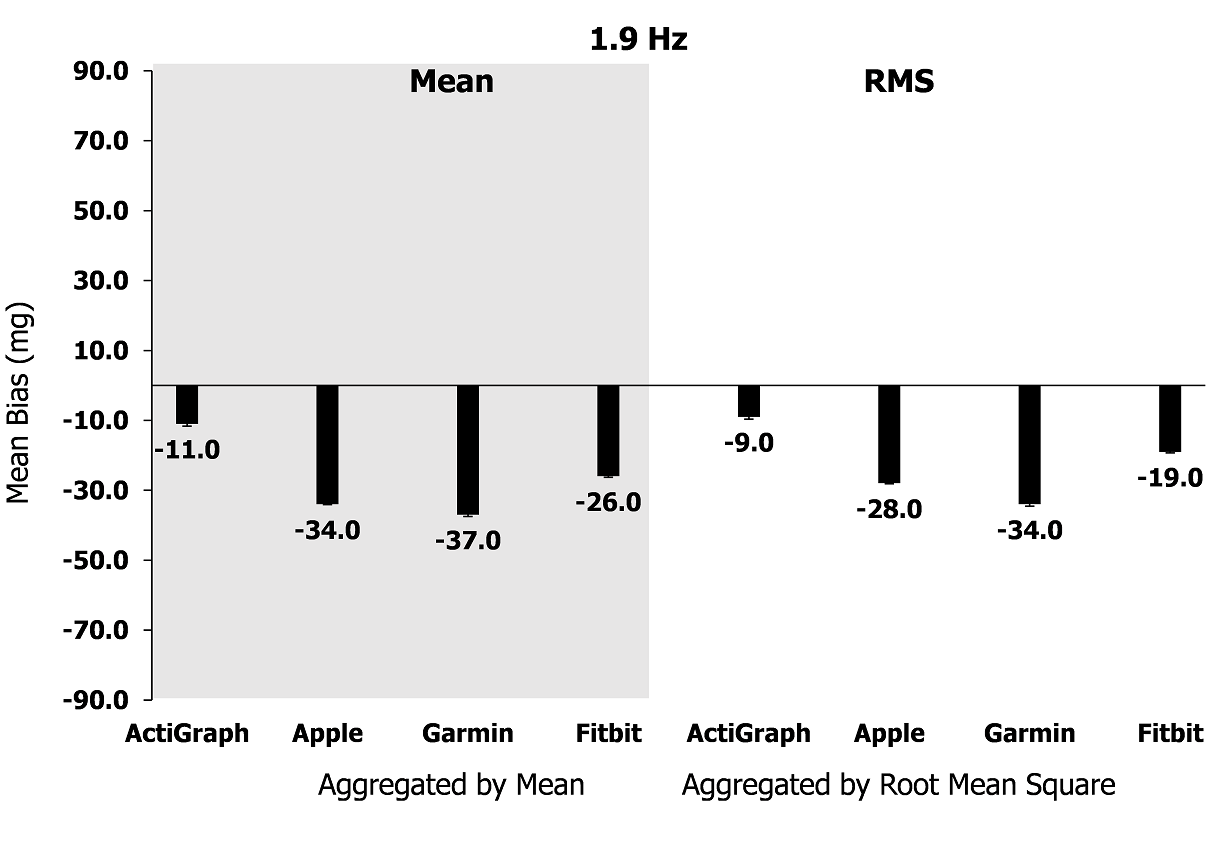 |
| e 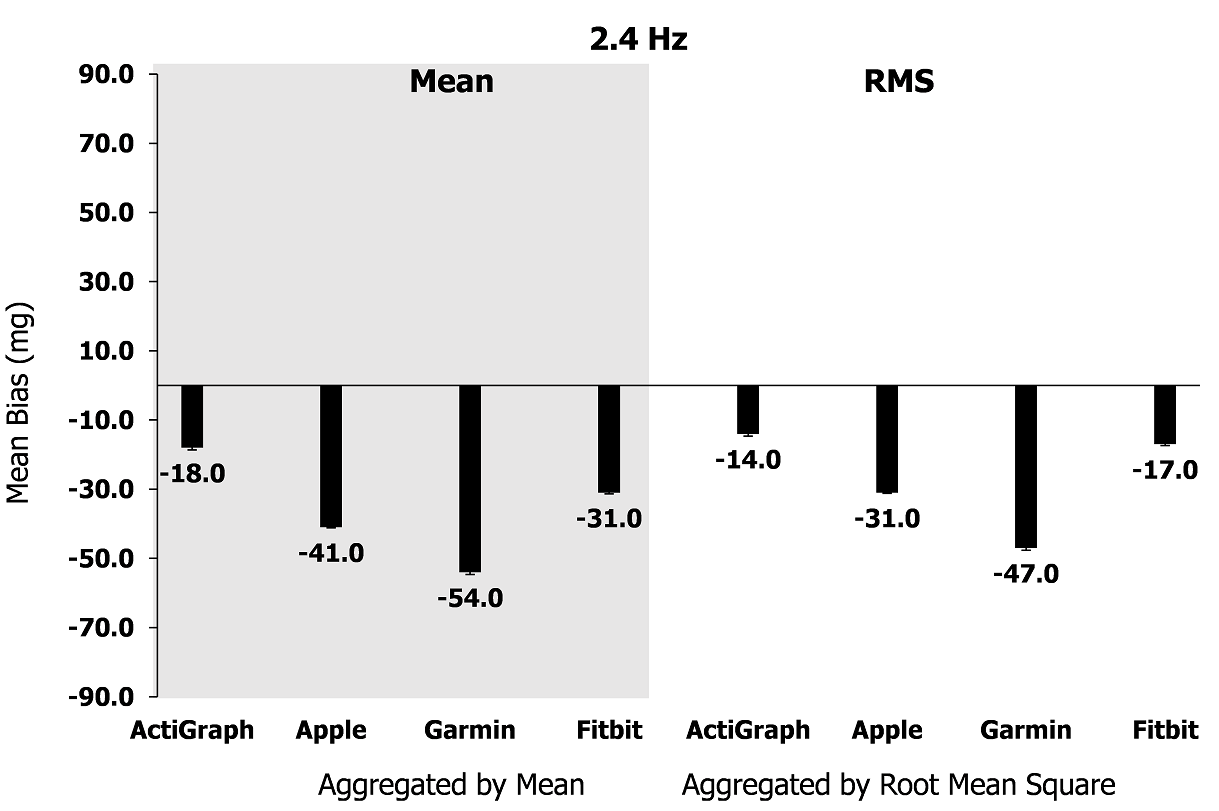 | f 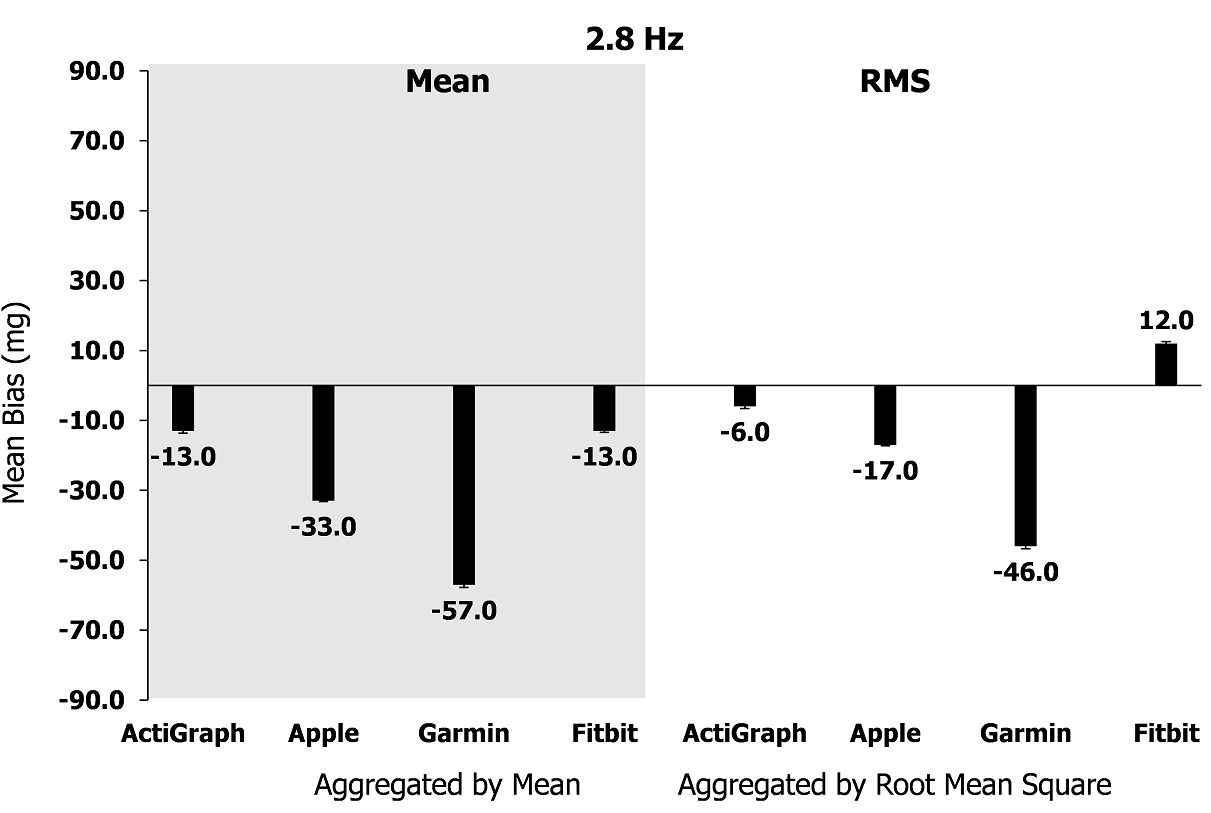 |
| g 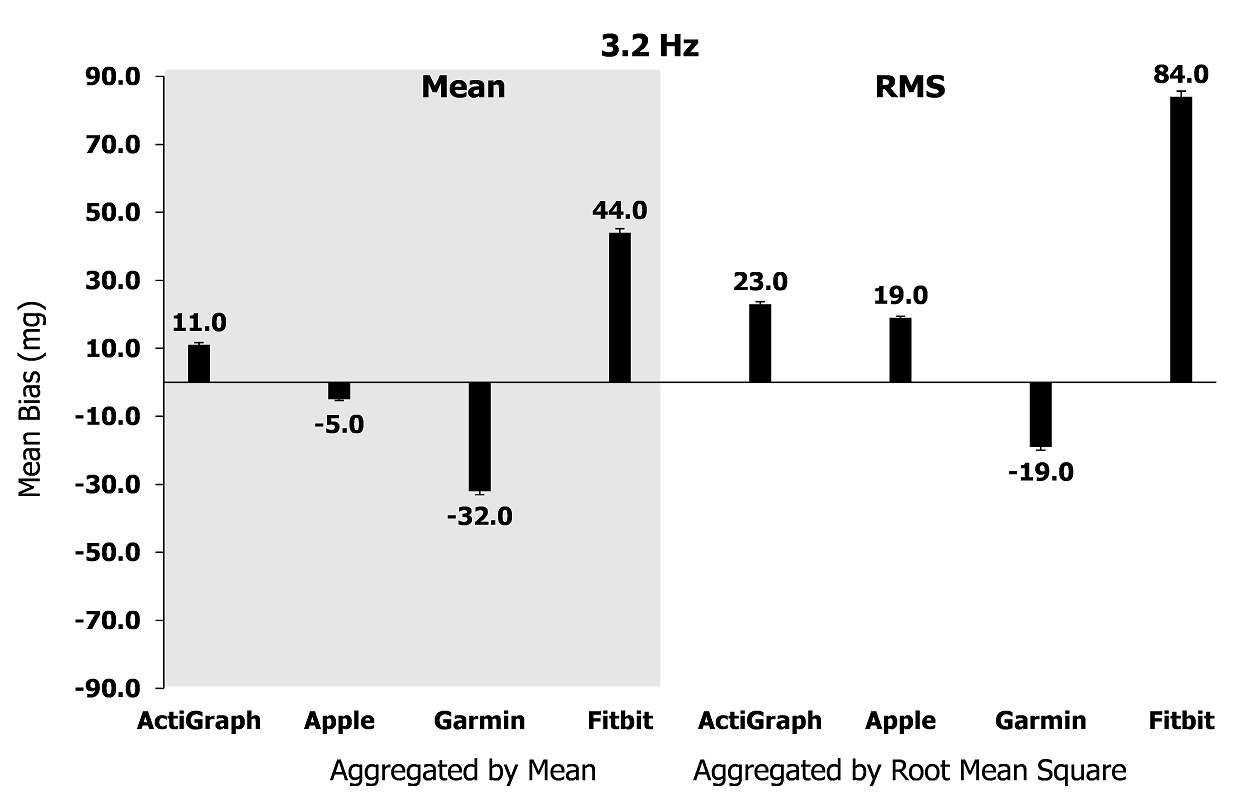 |

Supplement: S3 Fig — Error bars represent standard error. (DOCX) [file pone.0286898.s003.docx]
